# Genome Sequence Analysis of the Biogenic Amine-Degrading Strain *Lactobacillus casei* 5b

**DOI:** 10.1128/genomeA.01199-13

**Published:** 2014-01-16

**Authors:** Victor Ladero, Ana Herrero-Fresno, Noelia Martinez, Beatriz del Río, Daniel M. Linares, María Fernández, María Cruz Martín, Miguel A. Alvarez

**Affiliations:** Instituto de Productos Lácteos de Asturias, IPLA-CSIC, Villaviciosa, Asturias, Spain

## Abstract

We here report a 3.02-Mbp annotated draft assembly of the *Lactobacillus casei* 5b genome. The sequence of this biogenic amine-degrading dairy isolate may help identify the mechanisms involved in the catabolism of biogenic amines and perhaps shed light on ways to reduce the presence of these toxic compounds in food.

## GENOME ANNOUNCEMENT

*Lactobacillus casei* is a species of lactic acid bacteria (LAB) that is encountered in several habitats, including human mucosae and raw and fermented foods, especially dairy foods, in which it is the dominant nonstarter LAB during ripening (1). Such a varied ecological distribution has fueled the development of biotechnological applications for this microorganism, e.g., as probiotics (2), mucosal biotherapeutic agents (3), and detoxifiers of biogenic amines (BA) in cheeses (4).

BA are toxic compounds formed via the microbial decarboxylation of amino acids (5); they may accumulate in foodstuffs. The ingestion of foods with high BA concentrations leads to symptoms of intoxication (6). There is a general consensus regarding the need to reduce their presence in foods. Knowledge of the genome sequence of the BA-degrading strain *L. casei* 5b might provide insight into how this organism can be used as natural bioremediator.

We report here the draft genome of *L. casei* 5b, a strain isolated from cheese during a survey for BA-degrading LAB (4). The total DNA was extracted and a 0.5-kbp genomic library constructed and subjected to 90-bp paired-end sequencing (providing approximately 50-fold coverage) using a HiSeq 1000 system sequencer (Illumina) (performed at the Beijing Genomics Institute, China). Quality-filtered reads were assembled using Velvet software (http://www.ebi.ac.uk/~zerbino/velvet/), resulting in 91 contigs ranging from 208 to 411,480 bp. The total sequence length is 3,019,931 bp and the G+C content is 46.3%, a figure within the range of those of other *L. casei* genomes (46.1 to 46.6%). Annotation was performed using the Prokaryotic Genomes Annotation Pipeline (PGAAP) application on the NCBI server (http://www.ncbi.nlm.nih.gov/genomes/static/Pipeline.html) and was improved using the results obtained in a BLAST analysis (http://blast.ncbi.nlm.nih.gov). The genome contains 2,945 predicted coding sequences. Predicted copies of the 16S, 23S and 5S rRNA genes were found, as well as 56 genes for tRNAs.

A lactose phosphotransferase operon (*lacXGEFDCBAR*) was detected that confers the ability to metabolize lactose, as well as 2 cell wall-associated proteases (PrtR) (proteins N422_11395 and N422_11400) involved in casein metabolism. These genes are useful to the strain when growing in milk. No evidence of virulence-related or antibiotic resistance genes was found, and no genes involved in BA production were detected. The genome did contain one copy of the plasmid replication genes *repB* (N422_03780) and *copG* (N422_03775). This agrees with the presence of a single plasmid, as previously reported (4).

A comparison with the genome of *L. casei* BL23 (7), a non-BA-degrading strain (4), revealed the presence of 306 genes specific to *L. casei* 5b, although most were annotated as hypothetical proteins. Three methyltransferase and six oxidoreductase genes that might be involved in BA degradation were identified. Also detected was the gene for the protein N422_00550, which is similar to a recently characterized multicopper oxidase in *Lactobacillus plantarum* that was able to oxidize BA in wine (8).

The sequence analysis of the *L. casei* 5b genome might help identify pathways involved in detoxification of BA, leading to the development of new applications that might improve food safety.

### Nucleotide sequence accession numbers.

The results of this whole-genome shotgun project were deposited in the DDBJ/EMBL/GenBank database under the accession no. AUYM00000000 (BioProject PRJNA213287). The version of the genome described here has the accession no. AUYM01000000.
